# Effect of COVID-19 on Key Performance Indicators of Spanish Professional Soccer League

**DOI:** 10.3390/jfmk9010035

**Published:** 2024-02-21

**Authors:** José Fernández-Cortés, Carlos D. Gómez-Carmona, David Mancha-Triguero, Javier García-Rubio, Sergio J. Ibáñez

**Affiliations:** 1 Training Optimization and Sports Performance Research Group (GOERD), Sport Science Faculty, University of Extremadura, 10003 Caceres, Extremadura, Spain; jfernandxb@alumnos.unex.es (J.F.-C.); jagaru@unex.es (J.G.-R.); sibanez@unex.es (S.J.I.); 2 Physical Education and Sports Department, Cardenal Spínola CEU Andalucía University, 41930 Bormujos, Sevilla, Spain

**Keywords:** game indicators, notational analysis, contextual factors, football

## Abstract

The unprecedented COVID-19 health crisis severely disrupted global sports in 2020, prompting lengthy suspensions followed by resumed competitions under abnormal behind-closed-doors conditions without fans. These disruptions necessitated tactical adaptations by coaches and teams, attempting to still achieve successful outcomes. This study investigates the pandemic’s impacts on performance metrics and indicators within Spanish professional soccer. Utilizing systematic notational analysis, 760 match cases from the 2019–2020 La Liga season were examined, comprising 27 matchdays from the pre-COVID context and 11 after resumption. Multivariate tests identified significant pre/post differences and interactions for various technical indicators including shots, cards, corners, and offside calls. The pandemic was associated with a reduction from 12 to just 5 identifiable playing styles, suggestive of increased conservatism featuring more passive play, limited attacking depth, and horizontal ball movement. Such tactical changes appear provoked by condensed fixture scheduling post-lockdown, the lack of supportive crowds, and compromised player fitness/recovery. By quantifying these COVID-precipitated changes, the analysis provides tangible evidence for coaches to make informed adjustments in training and preparation for functioning effectively in disrupted environments. The findings emphasize that versatility and flexibility will be vital to optimize performance during times of unprecedented uncertainty.

## 1. Introduction

Performance analysis has become integral for success in modern soccer, with teams utilizing the detailed quantification of match events to gain competitive advantages [1]. Ball-related statistics offer key performance indicators, including passes, shots, clearances, and ball recoveries, which are supplemented by factors like possession and territory gained [2,3]. Extensive game data allow coaches to objectively evaluate strategies, optimize preparation drills, and increase the likelihood of victories [4]. Such analytics were originally focused on retrospective evaluations but have evolved towards predictive techniques, using machine learning on accumulated big datasets to extract new performance insights [5]. For this purpose, standardized definitions enable comparison, while trained specialists conduct the systematic observation and analysis of indicators [6,7]. Then, the findings derived from notational analyses are integrated into tailored training regimes crafted based on the assessments of opposition strengths, weaknesses, and playing styles [8,9,10].

One of the most studied aspects in soccer performance is the home advantage phenomenon [9,11]. Many studies have concluded that this advantage exists due to several factors such as fan support [12,13], territoriality [14], familiarity with the playing field [15], or referee bias [16]. However, in 2020, the unprecedented COVID-19 pandemic profoundly disrupted global sports, prompting the suspensions of competitions worldwide, followed by cautiously resumed contests held under abnormal behind-closed-doors conditions without fans after months of inactivity [17], as well as modifications in the players’ lifestyle like nutrition and supplementation [18]. The lengthy halts led to fitness declines and match sharpness losses from the lack of real-game situations [19,20]. Resumption increased injury risks due to congested schedules [21]. Most prominently, the mandated lack of spectators fundamentally impacted longstanding home advantage phenomenon and removed the supportive presence of familiar crowds along with their energizing influence [22].

Consequently, these pandemic-induced disruptions created unprecedented uncertainty and necessitated tactical adaptations by managers and teams who were still attempting to achieve successful performance outcomes [23,24]. Recent research examined COVID-19’s effects on the home advantage and referee bias through yellow cards and fouls, finding decreases without spectators [22,25]. However, a knowledge gap persists around impacts on key performance indicators for notational analysis. While initial research has uncovered decreased home advantages when examining factors like referee decisions, fouls, and disciplinary cards without fans present [26,27], questions remain about alterations in indicators tied to technical aspects and playing styles [25,28].

Accordingly, this study aims to explore the research gap by analyzing key performance indicators to quantify tactical and technical changes in Spanish First Division soccer league. Comparing statistics from matches before and after the pandemic, across home and away teams with different results, will provide data-driven insights about required adjustments for optimal preparation and training. The central questions across the 760 La Liga match cases from before and after the pandemic shutdown are as follows: (1) what effects did the widespread COVID-19 outbreak have on key performance indicators and match events?; (2) how did the lack of spectators during behind-closed-doors pandemic matches impact tactical choices and quantified metrics?; and (3) what changes are evidenced through data between pre- and post-lockdown contests regarding playing styles, strategic decisions, and priorities?

To address these questions, the research design utilizes systematic notational analysis to extract observations and detailed performance statistics describing match actions [29]. Data were gathered from official summaries. Coding focused on balls recovered, passes, shots, possession, and other key aspects. After compiling indicators, multivariate tests identify significant pre/post differences and interactions. Classification tree modeling then divides matches into homogeneous outcome groups based on performance indicators that shifted between the pre-COVID and behind-closed-doors pandemic phases. These findings could help coaches to objectively adapt rather than speculate amidst unpredictable disruptions.

## 2. Materials and Methods

### 2.1. Design and Procedures

The current study utilizes a quantitative methodology and is categorized as an empirical analysis featuring original empirical data produced by the authors and framed within the objectivist epistemology. It is a descriptive study where data were collected via an arbitrary observation code using systematic observation and notational recording based on a pre-constructed arbitrary code with a descriptive goal. The research is classified as ex post facto provided it transpired in a natural context where the phenomenon materializes without interference from the researchers [30].

A single experienced observer collected the notational analysis data to ensure consistency in data recording and interpretation. While multiple observers can improve reliability, the availability of video recordings in this study allowed repeat viewings to verify observations. As recommended by Lupo et al. [30], intra-observer reliability was assessed by having the observer re-analyze a random subsample of 10 matches, with 95% agreement achieved on key variables.

Following this design, data were extracted from the official website and reference portal of LaLiga (first Spanish professional soccer division). Moreover, situational data including venue, COVID stage (pre/post), and inter-stage divergences were coded. Initial analysis identified performance metrics with suspected influence on match outcomes for documentation. These selected variables were then used for subsequent examination. The next phase detected significant pre/post-lockdown discrepancies across indicators using specialized tests. Finally, classification tree algorithms categorized matches into outcome-delineated groups, reflecting modeled gameplay shifts from the pandemic disruption.

### 2.2. Sample and Variables

The dataset totaled 760 cases from the 2019/20 La Liga season, including pre-pandemic (27 matchdays) and post-lockdown (11 matchdays) phases separated by a 3-month period. Every match across 38 rounds contributed two case entries per game, one per club. Statistics were compiled directly from La Liga’s official website (https://www.laliga.com/laliga-easports/ (accessed on 5 October 2020)) and validated on three supplementary platforms, i.e., (1) Flashscore.com, (2) Whoscored.com, and (3) Soccerway.com, to cross-verify accuracy.

Independent variables were matched in terms of outcome (win/draw/defeat), venue (home/away), and COVID stage (pre/post). The following were designated as dependent variables per FIFA standards: disciplinary cards (yellow/red), possession, shots (total/on-target/off-target), set pieces (free kicks/corners), offsides, saves, fouls suffered, attacks, dangerous attacks, and passes. All variables were defined according to previous soccer research [31].

### 2.3. Statistical Analysis

Initial descriptive analysis through contingency tables displayed variable distributions. Subsequently, a multivariate general linear model identified performance differences between independent factors and match indicators. Observed power calculations then determined Type II error odds, with reference values of 0–0.2 (low), 0.2–0.5 (moderate), 0.5–0.8 (high), and >0.8 (very high) statistical potency [32].

Ultimately, regression evaluations and classification plus regression tree modeling (CRT) divided data points into optimally homogeneous terminal clusters relative to the outcome variable. The CRT approach followed an automated binary splitting method to create branching choice nodes delineating matches into groups based on key performance indicators like shots, cards, and possession that were significantly different between pre/post-COVID phases. Pure terminal nodes featured uniform dependent values across all enclosed cases [32]. Software package IBM SPSS 26.0 facilitated analytics to compare pre-post-COVID models (SPSS Inc., Chicago, IL, USA).

## 3. Results

Table 1 presents descriptive statistics across the independent variables of COVID stage (pre/post), outcome (win/draw/loss), and venue (home/away) against dependent performance indicators like disciplinary cards, possession, shooting, set pieces, saves, turnovers, attacking play, and passing.

Table 2 displays inferential analytics between independent factors and game metrics. COVID phase showed variance in yellow cards, total shots, wayward shots, corners, and offsides. Outcome exhibited differentiation in yellow/red cards, total/on-target/off-target shots, saves, and passes. Venue featured distinctions in possession, all shot variants, corners, offsides, saves, and general/dangerous attacks. COVID–venue interactions produced differences in total shots, errant shots, corners, and threatening attacks. Only outcome–venue displayed significance in terms of red cards. No game factors substantially varied across COVID–outcome or the three-way interaction.

**Figure 1:**
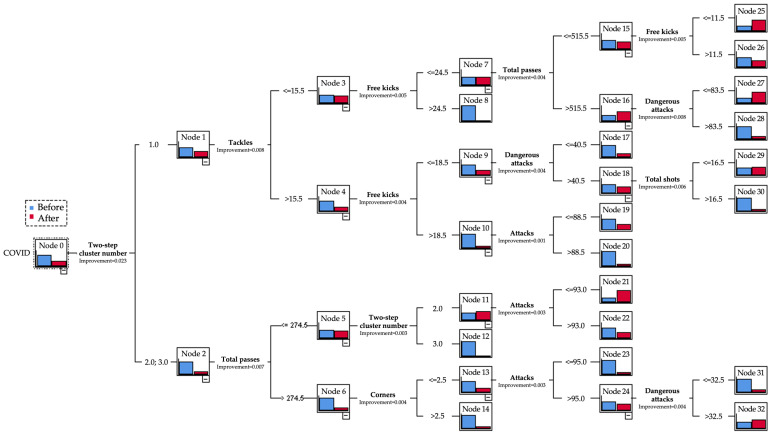
Graphical representation of the decision tree for the existing game systems in LaLiga.

The tree models in Figure 1 classify 12 pre-pandemic strategic styles as offensively aggressive, actively pressing, and exhibiting vertical possession and speedy counters/transitions. Five post-COVID styles emerge as more passive, defensively focused, horizontally possessive with minimal depth, and reliant on the central channel. As shown in Figure 1, pre-pandemic tactics appeared more offensively aggressive, exhibiting vertical possession, speedy counters, and active pressing. In contrast, the five pandemic systems seemed more conservative, featuring horizontal ball movement, passive play, and limited attacking committal.

## 4. Discussion

Quantitative match analysis is extensively used in team sports for performance enhancements in both research and practice [1,2,5,11,33,34]. Competition outcomes can be influenced by venue-based factors [9,35], spectator impacts [36], or scoring first [37]. The 2020 COVID-19 pandemic prompted the suspension of global sports leagues, leading to months of inactivity. Upon resuming under crowd-less conditions, this study examined whether deviations occurred in match metrics between pre- and post-lockdown contests in Spanish professional soccer. Therefore, the purpose of this investigation was to evaluate the role of match location, outcome, and the COVID-19 pandemic and the interplay between them regarding various key performance indicators. This investigation was conducted to determine if there were variances in the First Division soccer matches before and after the widespread outbreak.

The analysis revealed notable differences across several match performance indicators between the pre-pandemic and behind-closed-doors pandemic phases. Significant variance emerged for yellow cards, total shots, wayward shots, corners earned, and offside calls when examining the influence of COVID-19 disruption. Similarly, match outcome (win/draw/loss) produced differentiation in cards (yellows and reds), all shot types (total, on-target, off-target), saves made, and passes completed. Home advantage effects were present through metrics like possession percentage, shots, corners, attacking plays, and dangerous chances created. Additionally, changes in playing style diversity were evidenced by the reduction from 12 identifiably distinct systems pre-COVID to just 5 more homogeneous approaches post-pandemic.

### 4.1. Impact of COVID-19 on Match Indicators

The data showed considerable contrasts before and amid pandemic across factors like yellow cards, total attempts, off-target efforts, corners earned, and offsides called [28]. For yellows, observed power was very high (>0.8), while remaining metrics displayed good values (0.5–0.8). Aligning with these findings, Sors et al. found refereeing partiality shifted sans spectators [28]. Similarly, decreased shot volumes, especially lower quality off-target efforts, likely reflect the more conservative play calling by coaches and players without fans. Teams appeared reluctant to commit extra attackers forward to avoid being counterattacked if possession was lost. There was a clear air of risk aversion and reluctance to over-expose defensively.

The drop-off in set pieces like corners also indicates less overall attacking impetus or urgency to create dangerous chances. Similarly, fewer offside calls suggest more disciplined adherence to holding structured defensive shape rather than trying to spring early seeking goal-scoring opportunities. Ultimately, these metrics reflect increased conservatism and stagnancy, which are characteristics of the five post-COVID playing styles, showing limited attacking depth, reduced creativity, and reliance on horizontal ball circulation without attempts to unlock low blocks. The pandemic conditions seemingly provoked extreme risk aversion and reactive rather than proactive approaches [24].

Without supportive crowds, players reported feeling increased anxiety and pressure during matches, negatively influencing decisions [25]. The lack of fans also removed a key energy source that athletes draw from [22]. Consequently, the data imply players took fewer risks in attack without crowds to motivate elevated effort. The reduced attacking metrics correlated to compressed schedules and also hint that basic fitness and injury avoidance grew as priorities over entertainment or excitement.

### 4.2. Impact of COVID-19 on the Differences between Winning and Losing Teams

Additionally, notable variances emerged between winning, drawing, and losing clubs across yellows, reds, attempts (total, on goal, off goal), dives, and passes [1,3,10,38]. Reds, on-goal efforts, and off-goal attempts displayed very high observed power, while rest showed good levels. Backing these discoveries, Lago-Peñas et al. reported differences between winning and losing teams in shots [3]. Moreover, Červený et al. showed teams receiving cards in the World Cup had lower win probability [38]. Moreover, here, losing sides received more cards, especially reds, significantly cutting win chances without affecting referees. Furthermore, per Almeida et al., dives escalate among defeated teams, likely because weaker defense enables more opposition chances [10]. Finally, Liu et al. identified higher passes for winning versus losing clubs [1]. The literature agrees winning teams take more attempts and passes as they attack more, while struggling ones commit more cards and dives. Accordingly, victory requires more attempts and passes and less cards and dives; thus, coaches should develop aligned play styles with positive game indicator numbers.

### 4.3. Impact of Match Venue in the Result

The statistical analyses uncovered significant differences across variables pertaining to match location and the contextual factors of the COVID-19 pandemic and match results [1,23,24,39]. Specifically, in terms of the match venue-related metrics, good effect size values were observed for shots on-target, offsides calls, and dives committed, while possession percentage, total goal attempts, shots off-target, corner kicks, overall attacking plays, and dangerous scoring chances displayed very good effect sizes. These findings align with and support the work of Liu et al., who noted that home teams exhibited significantly higher mean values in possession and multiple shot categories (total, on goal, off goal) compared to those of visiting squads [1]. Home sides also recorded more overall attacking events and dangerous chances created from elements like corners and drawn offside calls versus their away opponents. Similarly, Antunez et al. also found that home teams tallied significantly more dives over the course of a season-long tournament, indicating an expansive offensive style when playing in front of their fans [39]. Consequently, based on these consistent venue-based effects, clubs and managers should strategically focus on elevating these beneficial game indicators when competing in home stadiums, while aiming to suppress these same statistical categories when playing visitor roles in away venues.

Additionally, with respect to the COVID-19 pandemic period, decreases were documented by both Almeida and Leite as well as Wunderlich et al. in total attempts, shots off-target, corners earned, and dangerous scoring chances created [23,24]. Although both home and away teams recorded declines, home squads underwent greater decreases, effectively reducing the typical home advantage as the German Bundesliga returned to action. While no current evidence describes or quantifies the specific influence of the pandemic on corners taken and dangerous attacks mounted, the overarching trends point to increased parity between home and away sides. As teams become more evenly matched in terms of venue-based performance effects, practitioners should shift towards training universal playing styles, tactics, and preparation methods suitable for consistent execution both at home stadiums and in away venues, with a focus on achieving positive numbers in the key game indicators that have been shown to contribute to victorious match outcomes.

Another factor to consider is playing style. As the gap between teams in the standings widens, their playing styles become more distinct. Bourbousson et al. [40] found that top teams exhibit more consistent and less context-dependent playing styles, while lower-ranked teams adapt their tactics more readily to the match situation [41]. The playing styles of weaker teams, particularly when faced with significant differences in rankings, are heavily influenced by their opponents (winners). This adaptability arises from the interplay between competing teams [42]. The diminished diversity of playing styles observed during the pandemic may be attributed to the reduced impact of situational factors such as the home advantage effect. Additionally, this period of competition coincided with a decline in athletes’ competitive readiness (three months without competitive matches) and increased fatigue due to the compressed match schedule (matches every 72 h). These factors contributed to a more cautious approach from teams, leading to the adoption of repetitive game strategies and a reluctance to take risks. This resulted in matches with lower scores and fewer goals. Teams in the German Bundesliga demonstrated a reduction in various game indicators when playing at home during the pandemic, suggesting a decrease in the significance of the home advantage. As the competitive balance between teams narrowed, they adopted more conservative playing styles [23,24].

### 4.4. Limitations and Future Research

While this research presents important initial evidence regarding the effect of the COVID-19 pandemic on playing styles and key game indicators before and after the widespread outbreak, some limitations must be acknowledged. Chiefly, there is a current lack of existing literature examining such pandemic impacts across other major European leagues and international club competitions. Additionally, the number of matches analyzed during the pandemic outbreak timeline proved smaller than the pre-COVID-19 sample. Employing larger samples of games played with and without spectators would provide enhanced statistical power for identifying trends. Finally, the condensed fixture schedules and frequent match cadence during the pandemic phases seemingly prompted some teams to overtly modify their typical styles of play, limiting their capacity to showcase principal strengths and tactical approaches. Such deviations could also plausibly stem from increased player absences due to injuries or illness during high-frequency match clusters.

Further inquiries should aim to implement bigger datasets while also weighing the influence of supplementary factors like travel-related fatigue, referee tendencies, territorial crowd effects, and the challenges presented by congested fixture schedules. As more leagues resume play amid varying pandemic stages, amassing evidence across these contexts will facilitate increasingly nuanced understanding of how disruptions and uncertainty influence performance metrics and strategic decisions. Collectively, by revealing the significant effects of the pandemic on key indicators and playing trends in Spanish First Division, this research provides a framework for continuing investigation within the evolving landscape of European and global soccer that could include a more holistic approach, incorporating the differences between ages of players and the changes in their lifestyle such as eating habits [18].

## 5. Conclusions and Practical Applications

The analysis revealed significant changes in performance metrics and playing styles between the pre-pandemic and behind-closed-doors phases. While these findings reflect the meaningful effects of COVID-driven spectator exclusions, the variances should not be overstated solely as tactical adaptations. Team and player qualities influence indicators and outcomes irrespective of environmental factors. For example, decreased shots and dangerous attacks post-lockdown could reflect declines in skills and decision making after months of inactivity as much as strategic conservatism. Similarly, condensed fixture congestion may have independently impacted injury rates and the availability of key performers.

Nonetheless, the quantified differences across situational conditions provide initial evidence to guide informed adjustments around preparation, training load management, and strategic versatility. Specifically, the reduced attacking output without supportive crowds suggests managers may need to motivate players differently to overcome risk aversion. Similarly, condensed scheduling warrants closer workload monitoring to minimize fatigue and injury risk. Implementing this context-specific versatility in being able to shift playing personality and statistics based on variables like spectators and rest should be a key objective.

While the COVID-19 crisis has abated, these insights remain highly relevant as they examine home versus away distinctions more broadly. The pandemic merely created an unprecedented “laboratory” condition to analyze venue-related factors in isolation. Spectatorless matches could occur for reasons unrelated to health crises, and home/away contrasts persist. Consequently, evidence-based optimization across settings can enhance consistency and sustained success. Achieving tactical flexibility to perform effectively across diverse environments should stand as a vital contemporary priority in the modern game.

## Figures and Tables

**Table 1:** Descriptive results of the analyzed variables and the influence of match venue and the presence/appearance of COVID.

Performance	Match	After COVID-19			Before COVID-19		
Index	Location	Win	Draw	Lost	Win	Draw	Lost
**YC**	Home	2.31 ± 1.45	2.65 ± 1.52	3.02 ± 1.66	2.33 ± 1.69	2.83 ± 1.17	2.49 ± 1.65
	Away	2.74 ± 1.51	2.92 ± 1.57	2.74 ± 1.45	1.91 ± 1.31	2.63 ± 1.56	2.16 ± 1.31
**RC**	Home	0.05 ± 0.24	0.08 ± 0.31	0.20 ± 0.43	0.11 ± 0.31	0.00 ± 0.00	0.31 ± 0.67
	Away	0.12 ± 0.32	0.13 ± 0.34	0.14 ± 0.37	0.11 ± 0.32	0.07 ± 0.25	0.13 ± 0.34
**Possession**	Home	50.72 ± 10.75	51.65 ± 11.21	51.27 ± 10.45	54.33 ± 12.56	50.20 ± 12.05	50.66 ± 12.86
	Away	48.73 ± 10.45	48.35 ± 11.21	49.28 ± 10.74	49.34 ± 12.86	49.80 ± 12.05	45.67 ± 12.56
**TS**	Home	13.47 ± 5.04	12.77 ± 4.85	12.21 ± 4.95	12.20 ± 3.48	10.10 ± 4.27	11.17 ± 3.78
	Away	10.65 ± 3.17	9.75 ± 4.31	9.92 ± 4.27	10.69 ± 3.99	9.77 ± 3.43	10.49 ± 4.75
**SoG**	Home	5.58 ± 2.62	3.80 ± 1.90	3.08 ± 2.05	5.07 ± 2.25	3.57 ± 1.67	3.09 ± 1.90
	Away	4.74 ± 1.76	3.13 ± 1.57	2.95 ± 1.76	4.97 ± 2.26	3.03 ± 1.29	3.11 ± 2.13
**SoffG**	Home	7.88 ± 3.62	8.97 ± 4.05	9.14 ± 4.12	7.13 ± 3.02	6.53 ± 3.50	8.09 ± 2.94
	Away	5.91 ± 2.79	6.61 ± 3.65	6.98 ± 3.48	5.71 ± 2.75	6.73 ± 3.34	7.38 ± 3.81
**FK**	Home	14.92 ± 4.07	16.36 ± 4.99	16.11 ± 4.30	14.24 ± 4.16	14.47 ± 3.70	15.46 ± 3.89
	Away	15.70 ± 4.46	15.68 ± 4.60	15.95 ± 4.67	15.14 ± 3.94	16.93 ± 5.07	14.96 ± 3.80
**Corners**	Home	5.02 ± 2.40	5.49 ± 2.90	5.65 ± 2.58	4.38 ± 2.85	4.17 ± 2.54	4.66 ± 2.90
	Away	4.12 ± 1.95	4.07 ± 2.33	4.23 ± 2.39	3.80 ± 2.12	4.33 ± 2.67	4.67 ± 2.54
**Offsides**	Home	2.40 ± 1.92	2.39 ± 1.89	2.50 ± 1.85	2.07 ± 1.45	2.37 ± 1.60	1.80 ± 1.36
	Away	2.06 ± 1.79	2.28 ± 1.72	1.77 ± 1.62	2.00 ± 1.51	1.80 ± 1.21	1.60 ± 1.40
**Dives**	Home	2.40 ± 1.69	2.19 ± 1.39	2.68 ± 1.65	2.80 ± 1.94	2.10 ± 1.29	2.80 ± 1.95
	Away	2.53 ± 1.79	2.85 ± 1.90	3.27 ± 2.22	2.60 ± 1.78	2.63 ± 1.27	2.98 ± 1.92
**Fouls**	Home	13.81 ± 4.46	13.56 ± 4.20	13.55 ± 3.80	13.02 ± 4.10	14.77 ± 4.31	13.69 ± 4.15
	Away	14.26 ± 4.08	14.49 ± 4.21	13.60 ± 3.87	13.57 ± 3.72	13.13 ± 3.30	13.24 ± 3.92
**Attacks**	Home	108.22 ± 25.08	113.76 ± 24.34	116.30 ± 23.32	112.20 ± 26.22	110.40 ± 23.63	110.63 ± 26.34
	Away	99.47 ± 21.46	107.69 ± 20.01	102.74 ± 22.20	104.43 ± 22.55	109.90 ± 23.52	101.64 ± 21.31
**DA**	Home	49.91 ± 17.64	54.05 ± 21.26	52.21 ± 15.77	48.84 ± 17.99	46.60 ± 14.38	48.63 ± 17.38
	Away	40.18 ± 13.20	42.25 ± 14.70	42.05 ± 14.69	40.46 ± 14.68	47.87 ± 13.20	43.93 ± 16.18

**Note. IdJ**: game indicator; **YC**: yellow card; **RC:** red card; **Possession**: amount of time the team controls the ball (expressed in %); **Total Shots (TS)**: total number of shots taken by the team; **Shots on Goal (SoG)**: number of shots on goal by a team; **Shots off Goal (SoffG)**: number of shots off goal by a team; **Free Kicks (FK)**: shots taken from fouls by the opposing team; **Corners**: number of corner kicks by a team; **Offsides**: infraction committed by violating the offside rule; **Dives**: total number of dives made by the goalkeeper; **Fouls**: total number of fouls committed by a team; **Attacks**: attacks made by a team with ball possession in the midfield; and **Dangerous Attacks (DA)**: attacks made by the team while having ball possession in the opponent’s half (opposing field).

**Table 2:** Inferential results of the independent variables and game indicators.

	Game Indicators	F	Sig	Observed Power
COVID-19	YC	7.510	0.006 *	0.781
	Total Shots	4.099	0.043 *	0.525
	Shots off Goal	5.107	0.024 *	0.617
	Corners	4.434	0.036 *	0.557
	Offsides	4.423	0.036 *	0.556
Results	YC	4.211	0.015 *	0.739
	RC	7.233	0.001 *	0.935
	Total Shots	3.622	0.027 *	0.670
	Shots on Goal	62.103	0.000 *	1.000
	Shots off Goal	6.651	0.001 *	0.913
	Dives	3.864	0.021 *	0.700
	Total Pass	5.272	0.043 *	
Match Venue	Possession	10.112	0.002 *	0.888
	Total Shots	24.518	0.000 *	0.999
	Shots on Goal	5.088	0.024 *	0.615
	Shots off Goal	23.633	0.000 *	0.988
	Corners	11.434	0.001 *	0.922
	Offsides	5.775	0.016 *	0.670
	Dives	4.556	0.033 *	0.568
	Attacks	15.946	0.000 *	0.979
	Dangerous Attacks	29.569	0.000 *	1.000
COVID-19—Match Venue	Total Shots	6.765	0.009 *	0.738
	Shots off Goal	6.952	0.009 *	0.750
	Corners	7.429	0.007 *	0.777
	Dangerous Attacks	6.168	0.013 *	0.699
Results—Match Venue	RC	4.035	0.018 *	0.720

Note. * *p* < 0.05; **YC**: yellow card; **RC:** red card; **Possession**: amount of time the team controls the ball (expressed in %); **Total Shots**: total number of shots taken by the team; **Shots on Goal**: number of shots on goal by a team; **Shots off Goal**: number of shots off goal by a team; **Free Kicks**: shots taken from fouls by the opposing team; **Corners**: number of corner kicks by a team; **Offsides**: infraction committed by violating the offside rule; **Dives**: total number of dives made by the goalkeeper; **Fouls**: total number of fouls committed by a team; **Attacks**: attacks made by a team with ball possession in the midfield; and **Dangerous Attacks**: attacks made by the team while having ball possession in the opponent’s half (opposing field).

## Data Availability

All data were extracted from https://www.laliga.com/laliga-easports/ (accessed on 5 October 2020).
